# “On-Off” Thermoresponsive Coating Agent Containing Salicylic Acid Applied to Maize Seeds for Chilling Tolerance

**DOI:** 10.1371/journal.pone.0120695

**Published:** 2015-03-25

**Authors:** Yajing Guan, Zhan Li, Fei He, Yutao Huang, Wenjian Song, Jin Hu

**Affiliations:** Seed Science Center, College of Agriculture and Biotechnology, Zhejiang University, Hangzhou, P.R. China; Henan Agricultural University, CHINA

## Abstract

Chilling stress is an important constraint for maize seed establishment in the field. In this study, a type of “on-off” thermoresponsive coating agent containing poly (*N*-isopropylacrylamide-*co*-butylmethacrylate) (Abbr. P(NIPAm-*co*-BMA)) hydrogel was developed to improve the chilling tolerance of coated maize seed. The P(NIPAm-*co*-BMA) hydrogel was synthesized by free-radical polymerization of N-isopropylacrylamide (NIPAm) and butylmethacrylate (BMA). Salicylic acid (SA) was loaded in the hydrogel as the chilling resistance agent. SA-loaded P(NIPAm-*co*-BMA) was used for seed film-coating of two maize varieties, Huang C (HC, chilling-tolerant) and Mo17 (chilling-sensitive), to investigate the coated seed germination and seedling growth status under chilling stress. The results showed that the hydrogel obtained a phase transition temperature near 12°C with a NIPAM to MBA weight ratio of 1: 0.1988 (w/w). The temperature of 12°C was considered the “on-off” temperature for chilling-resistant agent release; the SA was released from the hydrogel more rapidly at external temperatures below 12°C than above 12°C. In addition, when seedlings of both maize varieties suffered a short chilling stress (5°C), higher concentrations of SA-loaded hydrogel resulted in increased germination energy, germination percentage, germination index, root length, shoot height, dry weight of roots and shoots and protective enzyme activities and a decreased malondialdehyde content in coated maize seeds compared to single SA treatments. The majority of these physiological and biochemical parameters achieved significant levels compared with the control. Therefore, SA-loaded P(NIPAm-*co*-BMA), a nontoxic thermoresponsive hydrogel, can be used as an effective material for chilling tolerance in film-coated maize seeds.

## Introduction

Maize (*Zea mays* L.) is one of the most important thermophilic crops and is vulnerable to low temperatures. Due to unstable temperatures in the early spring in China, crops are often subjected to sudden low temperatures after sowing. These low temperatures inhibit seed emergence and delay seedling establishment, resulting in significant yield reduction and poor seed quality [1].

Seed coating is one of the principal technologies used to enhance seed performance in the field. Beneficial chemicals for plant growth, such as fungicides, insecticides, fertilizers and growth promoters, are usually delivered through coating agents to protect seeds from diseases and pests and to enhance field seedling establishment [2]. In recent years, the planting proportion of coated maize seeds to uncoated maize seeds increased both in China and worldwide [3]. Therefore, the study of coated maize seeds with chilling resistance is valuable.

For traditional seed-coating agents, the effective chilling-resistant ingredients are usually immediately released after the coated seeds are sowed regardless of the environmental temperature. Because of the large release and loss of ingredients prior to the occurrence of low temperatures, the chilling resistance effect of traditionally coated seeds has been weak [4]. Therefore, control of the release time and the release rate of chilling resistance agents is necessary to improve maize chilling tolerance.

Thermoresponsive hydrogels based on stimuli-sensitive polymers can alter their structure and physical properties in response to external temperatures [5]. The most popular thermoresponsive polymer, poly (N-isopropylacrylamide) (PNIPAm), has been extensively studied as a carrier for controlled drug-delivery systems [6]. PNIPAm exhibits a sharp phase transition at 32°C in aqueous media [7, 8]. This phase transition temperature is referred to as the lower critical solution temperature (LCST). Below the LCST, the polymer chain is hydrated and adopts an extended coil conformation, whereas above the LCST, the polymer is dehydrated and adopts a globular conformation [5]. Correspondingly, thermoresponsive hydrogels exhibit a reversible phase transition (swelling-shrinking) in response to external temperature changes to realize the “on-off” regulation of entrapped drug release.

The application of temperature-responsive materials in coated maize seed for chilling resistance has not previously been reported. When the environmental temperature is below 10°C, maize seed germination and seedling growth are strongly inhibited [9]. With the purpose of effectively improving maize chilling resistance, the LCST of the hydrogel was set to 12°C to release the chilling-resistant agent, which was used as a “temperature switch”. Zhang (2010) reported that the LCST can be altered by adjusting the quantity of the hydrophilic and hydrophobic monomers and that the LCST of the temperature-sensitive hydrogel can be decreased by modifying the proportion of free-radical polymerization of NIPAm and butylmethacrylate (BMA) [10]. Therefore, a P(NIPAm-*co*-BMA) hydrogel with an LCST of 12°C was used as the release controller in the maize seed-coating agents in the present study.

Salicylic acid (SA) is widely regarded as an endogenous plant growth regulator because it plays a significant role in the signal transduction pathway of abiotic stresses in plants [11, 12]. Enhanced resistance to low temperatures as induced by exogenous SA-induced has been reported in plants such as maize, wheat, banana, cucumber, and rice [13–16]. However, the effect of SA loaded in thermally responsive hydrogels on maize chilling tolerance has not been previously reported.

Consequently, a P(NIPAm-*co*-BMA) hydrogel was prepared and its thermo-sensitive performance was determined in this study. SA was used as the chilling resistance agent in the P(NIPAm-*co*-BMA) hydrogel. The chilling resistance of maize seeds coated with the SA-loaded P(NIPAm-*co*-BMA) was evaluated.

## Materials and Methods

### Materials

Maize seeds cv. Huang C (HC, chilling tolerance) and Mo17 (chilling sensitive) were supplied by the Zhangye Seed Company, China. The coating agents (talc and bentonite) and the adhesive were provided by the Yunnan Provincial Academy of Tobacco Agricultural Sciences, China. N-Isopropyl acrylamide (NIPAm, monomer), butyl methacrylate (BMA, monomer), N, N'-methylenebis acrylamide (BIS, cross-linking agent) and 2, 2'-azobisisobutyronitrile (AIBN, initiator) were purchased from Aladdin Industrial Inc. Shanghai, China. Salicylic acid (SA, chilling resistance agent), 1, 4-dioxane, methanol, toluene and hexane were purchased from Shanghai Dingguo Biotech Co., Ltd, Shanghai, China.

### Methods

#### Synthesis of the P(NIPAm-*co*-BMA) hydrogel

First, NIPAm was crystallized from toluene/hexane [5], and BIS and AIBN were purified in methanol. Then, 0.977 g of NIPAm, 0.1942 g of BMA and 0.077 g of BIS were dissolved in 11 ml of 1, 4-dioxane in a glass tube. After bubbling nitrogen through the mixed solution for 5 min to remove the dissolved oxygen, 0.016 g of AIBN was added. Nitrogen was then bubbled into the solution for 15–20 min. Next, the glass tube was sealed and immersed in a 70°C water bath. The P(NIPAm-*co*-BMA) hydrogel was polymerized by the cross-linking of NIPAm and BMA after 24 h. Finally, the unreacted compounds and solvents were extracted by soaking the hydrogel in a water/methanol (50/50, v/v %) solution for one week, and the polymer was completely dried under vacuum for 7 d at 25°C.

#### Lower critical solution temperature determination of the P(NIPAm-*co*-BMA) hydrogel

A certain amount (W_0_) of dried hydrogel was immersed in water of different temperatures (a temperature interval of 3°C) to fully swell the gel. The weight of the fully swollen hydrogel (W_t_) in relation to its dried weight (W_0_) was defined as the swelling ratio (SR). The LCST was determined from the sharp change point in the curve of the SR vs. temperature.

#### Fourier transform infrared spectroscopy analysis and scanning electron microscope images of the P(NIPAm-*co*-BMA) hydrogel

In this step, 0.01 g of dried hydrogel was combined with a certain amount of KBr and ground into powder. The mixed powders were pressed into small discs under vacuum and analyzed by Fourier transform infrared spectroscopy (FTIR) using the absorbance mode. FTIR spectra were obtained in the wave number range from 4000 to 500 cm^−1^ (Nicolet 5700, Thermo Nicolet, USA). In addition, a sample of dried hydrogel was analyzed on a scanning electron microscope (SEM) to observe the apparent morphology.

#### Swelling measurements of the P(NIPAm-*co*-BMA) hydrogel

Dried hydrogels (*m*_*0*_, g) were swelled in distilled water at 5°C and were weighed every 5 h for a total of 30 h. The swelling ratio (SR) was calculated as $SR=\frac{m_{t}-m_{0}}{m_{0}}\times 100\text{\%}\;$(g/g), where *m*_*0*_ and *m*_*t*_ are the weights of the hydrogels in the dry and swollen state at each testing time, respectively. Each swelling ratio was an average of three samples, and the results are reported as the mean ± standard deviation.

The swelling ratio of the fully swollen hydrogel was defined as the equilibrium swelling ratio for each condition.

#### Water retention determination of the P(NIPAm-*co*-BMA) hydrogel

Dried hydrogels (*m*_*0*_, g) were swelled in distilled water at 5°C for 3 d. The fully swollen hydrogels (*m*_*e*_, g) were then immersed into 30°C water to deswell. The gels were weighed (*m*_*t*_, g) every 5 min. The water retention ratio (RR) at 30°C was calculated as $RR=\frac{m_{t}-m_{0}}{m_{e}-m_{0}}\times 100\text{\%}$ (g/g), where *m*_*0*_, *m*_*e*_ and *m*_*t*_ are the dried hydrogel weight, the initial equilibrium swollen hydrogel weight and the weight obtained for each testing time at a given temperature, respectively.

#### SA loading into the P(NIPAm-*co*-BMA) hydrogel

SA was loaded into the P(NIPAm-*co*-BMA) hydrogel by a solvent sorption method. SA (5 g) was dissolved in 100 ml ethanol/water (80/20, v/v %) solution. The dried hydrogel (*m*_*0*_, g) was immersed in a 5% (W/V) SA mixed solution for 3 d at 5°C. The residual SA on the hydrogel surface was rinsed by ethanol. Then, the SA-loaded P(NIPAm-*co*-BMA) swollen hydrogel was dried for 5 d under vacuum, weighed and ground into powder for further study. The SA loading ratio (W_SA_) was determined by $W_{SA}=\frac{m_{t}-m_{0}}{m_{0}}\times 100\text{\%}$ (g/g), where *m*_*0*_ and *m*_*t*_ are the weights of the initial dried hydrogel and the SA-loaded dried hydrogel, respectively. The SA loading ratio was 47.5% and the SA loaded P(NIPAm-*co*-BMA) hydrogel was used for further experiments.

#### SA release from the P(NIPAm-*co*-BMA) hydrogel

Two SA release experiments were conducted:

1. To test the SA release from the hydrogel under a thermal cycling operation between 9°C and 15°C, a certain amount of dried SA-loaded P(NIPAm-*co*-BMA) hydrogel was placed in 10 ml ddH_2_O. The solution was gently stirred, and a 1-ml sample was withdrawn (replaced by 1 ml of ddH_2_O) every 30 min. The water temperature oscillated between 9°C and 15°C every hour.
2. To test the pulsatile SA release from the hydrogel under different pH values, a certain amount of dried SA-loaded P(NIPAm-*co*-BMA) hydrogel was placed in phosphate buffer solutions (PBS) with different pH values (pH = 5, 7, 9). The PBS temperature oscillated between 9°C and 15°C every 15 min. Samples were taken every 15 min.

The SA concentrations of all samples were monitored at 296 nm on a UV spectrophotometer (UV-2450, Shimadzu, Japan).

#### Preparation of coated maize seeds

Seven seed-coating agents were prepared in this study according to the formula given in Table 1. Maize seeds were coated with an adhesive and the coating agents (seeds: coating agents = 5:4 (w/w)) in a cyclic alternating pattern using a ‘‘BY300A” minitype coater (Shanghai, China). Firstly, 50 g of maize seeds were put into rotating pan of 300mm diameter with 40^o^ of inclination and 55 rpm of pan speed. Secondly, 2.0 ml of adhesive were sprayed onto the seed surface, and 10 g of prepared coating agents (see the Table 1) were added into the pan for 1 min rolling. Repeat the secondly step twice. Finally, 3 ml of adhesive were sprayed onto the seed surface for 5 min rolling. The coated seeds were then air-dried for 2 d at room temperature. Coated seeds containing neither P(NIPAm-*co*-BMA) nor SA were used as a control.

**Table 1:** Formulas of the maize seed-coating agents.

Formula*	P(NIPAm-*co*-BMA) (g)	SA (g)	SA-loaded P(NIPAm-*co*-BMA) (g)	Talc (g)	Bentonite (g)	Total weight (g)
0	0	0	-	70.00	30	100
1	0.1	0	-	69.90	30	100
2	1.0	0	-	69.00	30	100
3	0	0.05	-	69.95	30	100
4	0	0.50	-	69.50	30	100
5	-	-	0.15	69.85	30	100
6	-	-	1.50	68.50	30	100

*P(NIPAm-*co*-BMA) is poly (N-isopropyl acrylamide-*co*-butylmethacrylate) and SA is salicylic acid; **formula 0** is the mixture of talc and bentonite without P(NIPAm-*co*-BMA) or SA; **formulas 1** and **2** are mixtures of P(NIPAm-*co*-BMA), talc and bentonite without SA; **formulas 3** and **4** are mixtures of SA, talc and bentonite without P(NIPAm-*co*-BMA); **formulas 5** and **6** are mixtures of SA-loaded P(NIPAm-*co*-BMA), talc and bentonite. In addition, for formula 5, 1.05 g of SA-loaded P(NIPAm-*co*-BMA) contained 0.1 g of P(NIPAm-*co*-BMA) and 0.05 g of SA; for formula 6, 1.50 g of SA-loaded P(NIPAm-*co*-BMA) hydrogel contained 1 g of P(NIPAm-*co*-BMA) and 0.50 g of SA.

#### Measurement of seed germination and seedling quality under chilling stress

Following seed coating, a seed germination test was performed. Fifty seeds for each treatment were placed in a plastic germination box (12 cm×18 cm) with a sand bed, and each experiment was replicated four times. The seeds were incubated in a germination chamber at 25°C under an alternating cycle of 12 h of illumination and 12 h of darkness for 5 d [17] and then subjected to a low temperature stress at 5°C for 3 d. Next, the seedlings were transferred to 25°C to recover growth for 3 d. Germinated seeds were counted daily, and the germination energy (GE) and germination percentage (GP) was calculated on the 5^th^ and 11^th^ day, respectively. The germination index (GI) was calculated according to Hu *et al*. (2005) [18]:GI=∑Gt/Tt, where *Gt* is the number of new germination seeds in time *Tt*. The root length (RL) and shoot height (SH) were measured manually with a ruler, and the root and shoot dry weight (RDW and SDW) were determined after drying at 80°C for 24 h [19]. These measurements were conducted on thirty randomly selected normal maize seedlings for each replicate.

#### Investigation of physiological changes in maize seedlings under chilling stress

The peroxidase (POD) and ascorbate peroxidase (APX) activities in seedling shoots and roots were determined by the method described by Qiu *et al*. (2005) [20]. Seedling catalase (CAT) and superoxidase dismutase (SOD) activities were measured by the method described by Cao *et al*. (2010) [21]. The malondialdehyde (MDA) content in seedling shoots and roots was measured using the thiobarbituric acid reaction method [22]. All of the above measurements were conducted after a seed germination duration of 11 d. An absorbance change of 0.01 in 1 min is defined as 1 unit of enzyme activity (U): U/g·min·FW.

#### Statistical analysis

Statistical analyses of all data were performed using analysis of variance (ANOVA) with SAS version 8.0 software (SAS Institute, Cary, NC). Fisher’s least significant difference (LSD) tests (α = 0.05) were adopted for multiple comparisons. The percent data were transformed according to y = arcsin [sqr (x/100)] before ANOVA.

## Results

### Characterization of the P(NIPAm-*co*-BMA) hydrogel

#### Morphological characterization of the P(NIPAm-*co*-BMA) hydrogel

**Fig 1:**
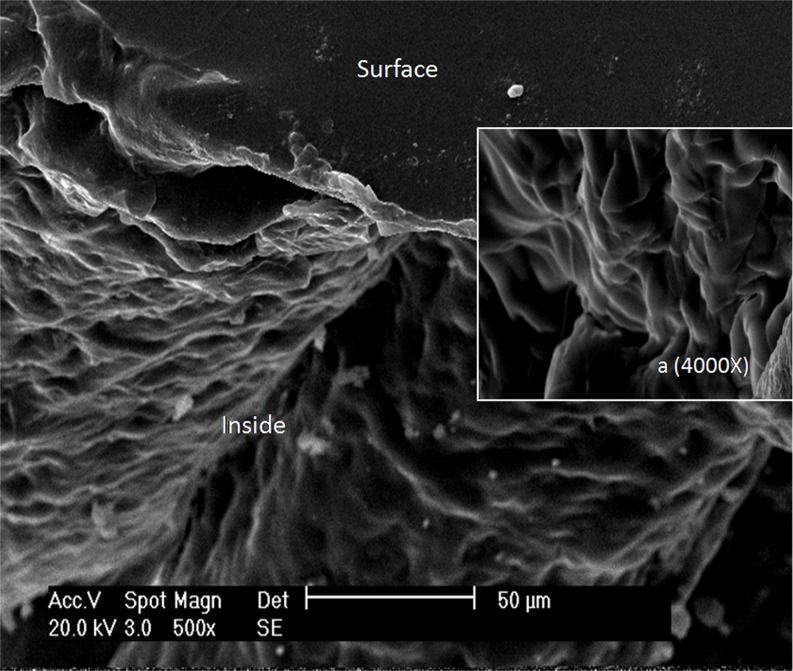
Scanning electron microscopy images of the P(NIPAm-*co*-BMA) hydrogel (×500). P(NIPAm-*co*-BMA) is poly (N-isopropyl acrylamide-*co*-butylmethacrylate); ‘Surface’ and ‘Inside’ indicates the surface and inside of P(NIPAm-*co*-BMA) hydrogel, respectively. ***a***, the inside of P(NIPAm-*co*-BMA) hydrogel (**×**4000).

Fig. 1 shows the SEM images of the dried P(NIPAm-*co*-BMA) hydrogel. Abundant thin-film micropores were detected inside the hydrogel, indicating that the hydrogel had a relatively loose structure. This loose structure promotes a good swelling/deswelling ratio to respond to lower/higher system temperatures in comparison to the LCST. The micropores could be observed more clearly under 4000x magnification (Fig. 1a). The surface of the hydrogel was slightly more compact than its interior.

#### Temperature-responsive characteristics of the P(NIPAm-*co*-BMA) hydrogel

**Fig 2:**
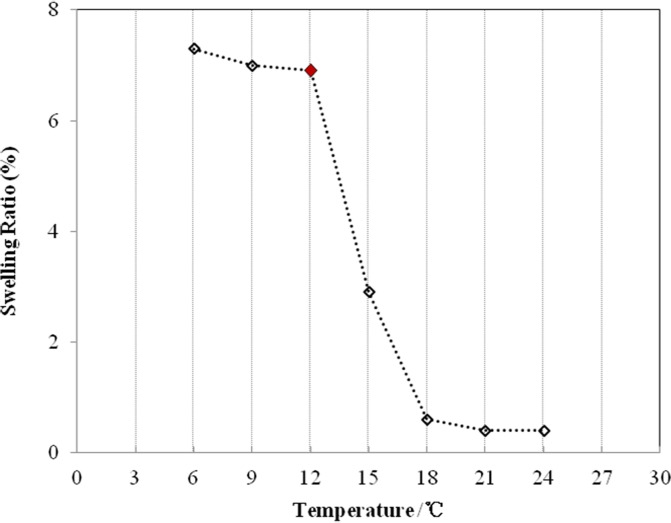
The lower critical solution temperature (LCST) profile of P(NIPAm-*co*-BMA) hydrogel. P(NIPAm-*co*-BMA) is poly (N-isopropyl acrylamide-*co*-butylmethacrylate). The LCST was determined from the sharp change point (indicated by red diamond) in the curve of the swelling ratio vs. temperature.

The SR of the P(NIPAm-*co*-BMA) hydrogel changed sharply at 12°C (Fig. 2), which was considered as the LCST of the P(NIPAm-*co*-BMA). When the temperature was below 12°C, the hydrogel had a higher SR compared to that above 12°C.

**Fig 3:**
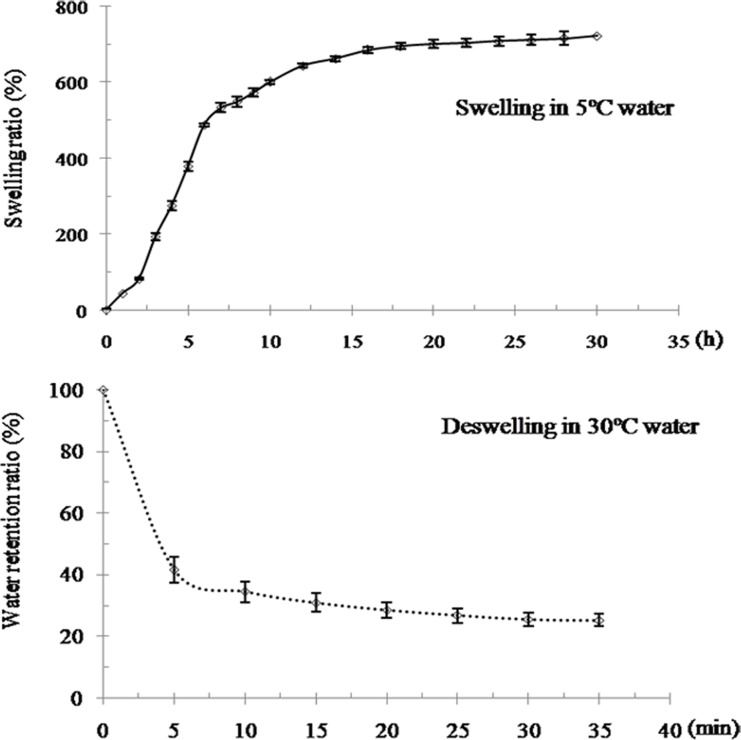
The swelling and deswelling characteristics of the P(NIPAm-*co*-BMA) hydrogel. P(NIPAm-*co*-BMA) is poly (N-isopropyl acrylamide-*co*-butylmethacrylate). The swelling characteristic is determined by the swelling ratio of P(NIPAm-*co*-BMA) hydrogel in distilled water at 5°C; the deswelling characteristic is determined by the water retention ratio of P(NIPAm-*co*-BMA) hydrogel in distilled water at 30°C. Error bars represent ±S.E.

Fig. 3 shows swelling and deswelling measurements obtained at 5°C and 30°C, respectively. The SR and RR of the hydrogel were calculated for each testing time until the weight of the hydrogel remained constant. After swelling in water at 5°C for 5 h, the SR exceeded 300%; this value reached 700% at 30 h. The fully swollen hydrogel quickly lost water at 30°C and retained only a quarter of the maximum water content after a deswelling duration of 30 min.

### Characterization of the SA-loaded P(NIPAm-*co*-BMA) hydrogel

#### Fourier transform infrared spectroscopy analysis of P(NIPAm-*co*-BMA) and SA-loaded P(NIPAm-*co*-BMA) hydrogels

**Fig 4:**
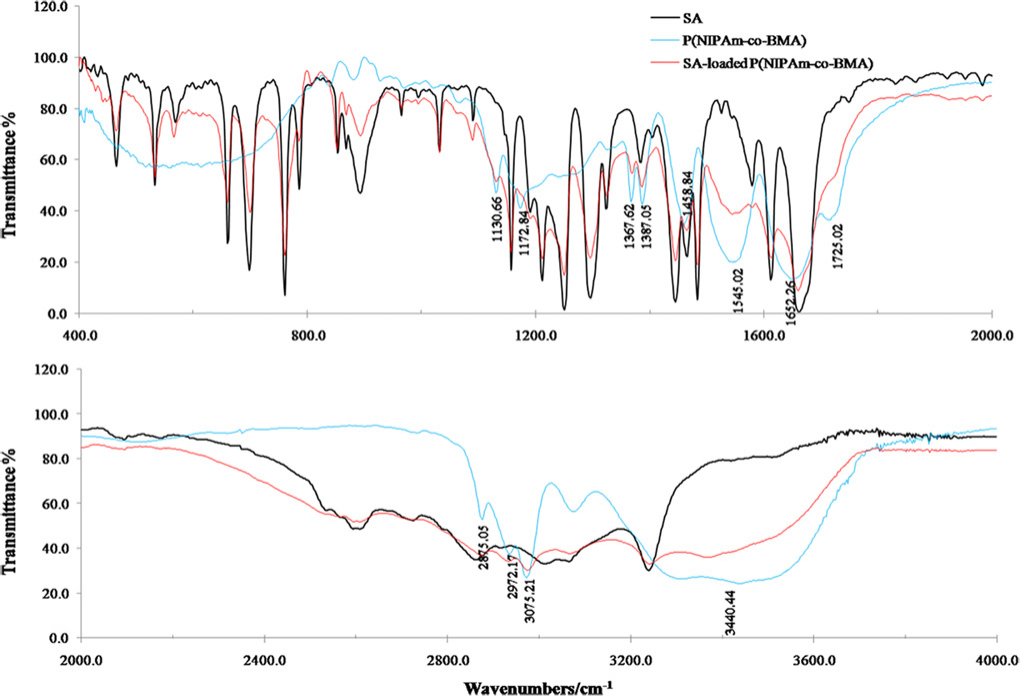
The fourier transform infrared spectroscopy (FTIR) spectrum of SA, the P(NIPAm-*co*-BMA) hydrogel and the SA-loaded P(NIPAm-*co*-BMA) hydrogel. P(NIPAm-*co*-BMA) is poly (N-isopropyl acrylamide-*co*-butylmethacrylate) and SA is salicylic acid; The SA-loaded P(NIPAm-*co*-BMA) hydrogel is the P(NIPAm-*co*-BMA) hydrogel loaded by SA through a solvent sorption method; The absorption peaks showed in the figure indicate special chemical bonds or functional groups. The top figure is the FTIR spectrum from 400–2000 cm^-1^ wavenumbers and the bottom figure is the FTIR spectrum from 2000–4000 cm^-1^ wavenumbers.

A typical absorption spectrum of the P(NIPAm-*co*-BMA) hydrogel is shown in Fig. 4. The characteristic stretching peaks of the NIPAm units were observed at 3440.44 cm^-1^ and 3075.21 cm^-1^, resulting from the N-H group. A vibration absorption peak was observed at 2870–2975 cm^-1^due to the C-H groups of methyl, methylene and methyl on the saturated-hydrocarbon main chain. The amide I peak at 1652.26 cm^-1^ (C = O stretching) and the amide II peak at 1545.02 cm^-1^ (N-H bending vibration and C-N stretching) were attributed to the amide bond of NIPAm. The-CH (CH_3_)_2_- vibration absorption peaks were observed at 1458.84 cm^-1^, 1387.05 cm^-1^and 1367.62 cm^-1^, and the peaks at 1387.05 cm^-1^and 1367.62 cm^-1^ indicated similar symmetric rotor methyl CH-stretch vibrations. The characteristic peaks of BMA were observed at 1725.02 cm^-1^ (C = O stretching), 1172.84 cm^-1^ and 1130.66 cm^-1^ due to the C-O-C group (asymmetric stretching).

After SA was loaded into the hydrogel, the characteristic stretching peak of the NIPAm units remained at 3440 cm^-1^, arising from the specific N-H group, and the amide II peak remained at 1544 cm^-1^ because of N-H bending and C-N stretching vibration (Fig. 4). However, several specific vibration absorption peaks of P(NIPAm-*co*-BMA) weakened due to the influence of the C-H and C = C bonds of the SA.

#### SA release from the P(NIPAm-*co*-BMA) hydrogel

**Fig 5:**
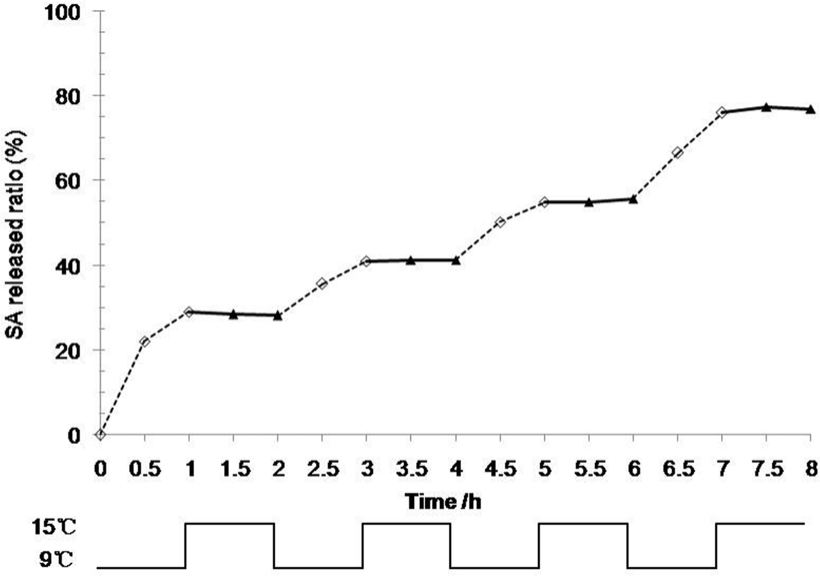
Effect of circulative pulse temperatures on SA accumulative release from the P(NIPAm-*co*-BMA) hydrogel. P(NIPAm-*co*-BMA) is poly (N-isopropyl acrylamide-*co*-butylmethacrylate) and SA is salicylic acid. SA release ratio from P(NIPAm-*co*-BMA) hydrogel is indicated by hollow diamond under 9°C (◇) and by solid triangle under 15°C (▲). Temperature changes between 9°C and 15°C in the form of circulative pulse. Statistical error bars are not easy to see because they are covered by the symbols.

A ladder-like SA release curve is depicted in Fig. 5. SA was released at a high rate at 9°C when the hydrogel was swelling; however, the SA release slowed quickly at 15°C when the hydrogel was deswelling. The slope of the SA release curve was greater at 9°C than at 15°C (Fig. 5).

**Fig 6:**
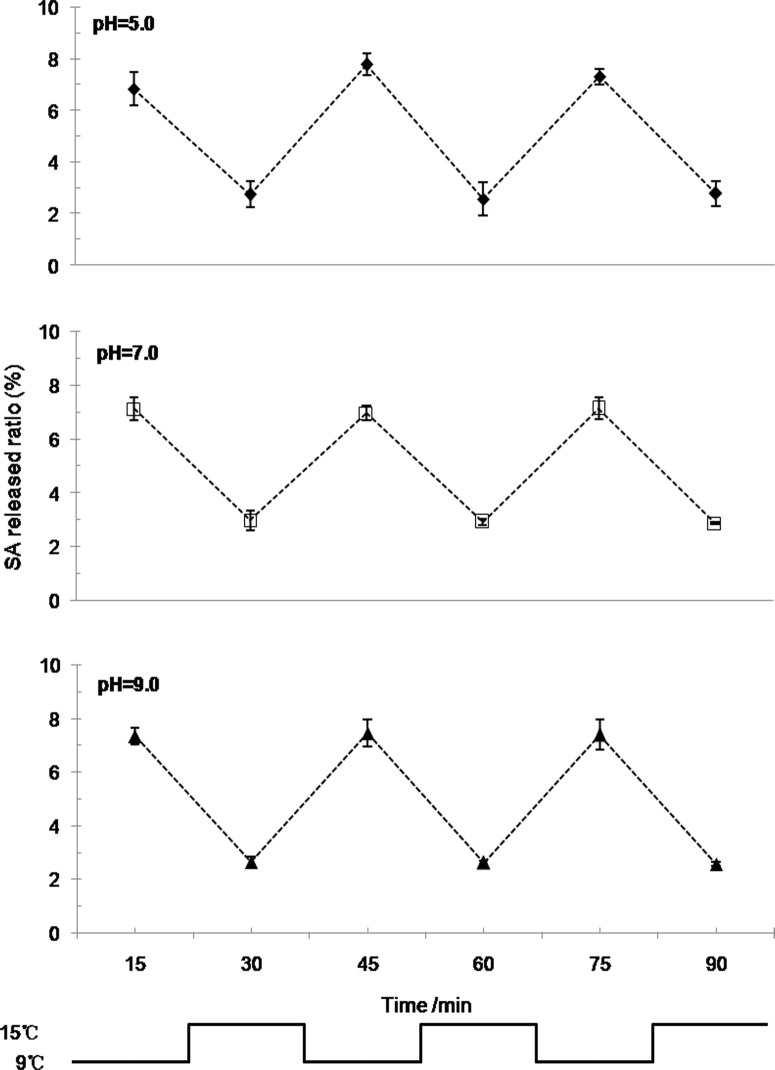
The effect of pH on SA release from the P(NIPAm-*co*-BMA) hydrogel under circulative pulse temperatures. P(NIPAm-*co*-BMA) is poly (N-isopropyl acrylamide-*co*-butylmethacrylate) and SA is salicylic acid. The pulsatile SA releases from the hydrogel under phosphate buffer solutions with different pH values (pH = 5, 7, 9). The circulative pulse temperature changes between 9°C and 15°C every 15 min. Error bars represent ±S.E.

In addition, the SA release ratio patterns under circulative pulse temperatures in three PBS buffers with different pH values were similar (Fig. 6). Almost 7.0% of the SA was released from the P(NIPAm-*co*-BMA) hydrogel every 15 min at 9°C; however, only 3.0% of the SA was released at 15°C.

### Effects of coating agents containing SA-loaded P(NIPAm-*co*-BMA) on maize seed germination and seedling growth under chilling stress

For both maize varieties, no significant differences in seed germination among J1, J2 and the control were detected. S1 improved the GE, GP and GI under chilling stress compared with the control; however, none of the parameters achieved significant levels (Table 2). S2 had a higher SA concentration and significantly inhibited seed germination. J1S1 improved the GE, GP and GI of HC and Mo17, and the increased GP of Mo17 achieved a significant level. Except for the GE of Mo17, the seed germination parameters in both maize varieties treated by J2S2 were significantly higher than those of the control, J1 and S2.

**Table 2:** Effect of coating agent on maize seed germination under chilling stress.

Treatment	HC			Mo17		
	GE (%)	GP (%)	GI	GE (%)	GP (%)	GI
Control**	68.58±1.64bc*	71.29±3.23bc	7.56±0.61b	48.57±3.30ab	53.38±3.43c	5.11±0.34b
J1	60.95±2.52c	66.67±4.16c	7.27±0.21b	43.81±4.15b	53.33±2.52c	4.96±0.27b
J2	60.95±2.52c	65.71±1.65c	7.11±0.19b	44.76±2.52b	54.28±1.65c	5.14±0.21b
S1	71.43±5.72abc	79.05±1.90ab	7.65±0.70b	53.33±2.2a	60.00±1.65bc	5.97±0.17b
S2	26.67±5.30d	30.48±4.78d	2.69±0.43c	27.62±0.95c	37.14±3.30d	3.13±0.12c
J1S1	75.24±5.04ab	75.24±5.04abc	7.93±0.20b	51.43±3.30a	65.7±4.36ab	6.02±0.07b
J2S2	80.95±1.90a	84.76±1.90a	9.27±0.15a	53.33±0.95a	70.48±3.43a	6.25±0.27a

GE, germination energy; GP, germination percentage; GI, germination index; HC, Huang C.

*Significant difference (α = 0.05, LSD) among treatments within the same variety.

**Control. maize seeds coated with formula 0; **J1**. maize seeds coated with formula 1; **J2**. maize seeds coated with formula 2; **S1**. maize seeds coated with formula 3; **S2**. maize seeds coated with formula 4; **J1S1**. maize seeds coated with formula 5; **J2S2**. maize seeds coated with formula 6. The coating agent was applied at a ratio of 1 g to 5 g of naked seeds for all of the treatments. The different formulas are shown in Table 1.

**Fig 7:**
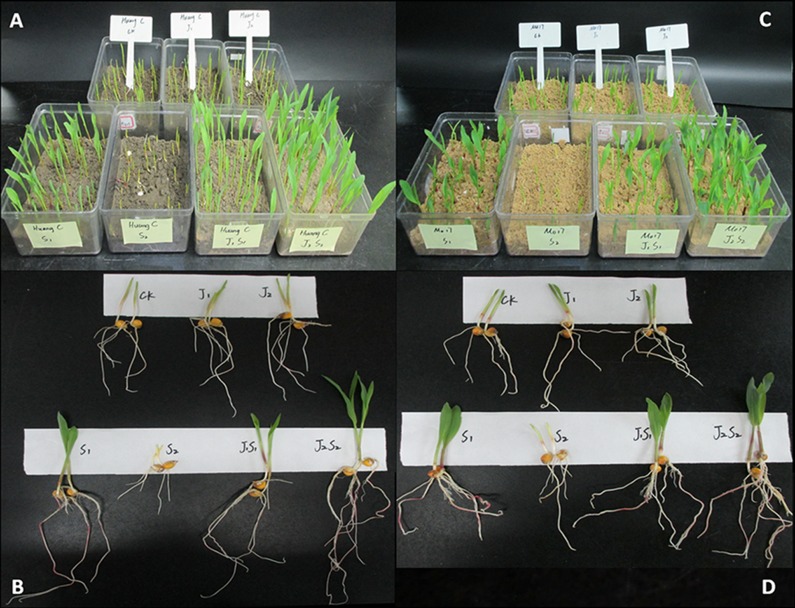
Effect of agent on seedling growth of maize varieties Huang C (HC) and Mo17 after chilling stress. (**A** and **B** are Huang C; **C** and **D** are Mo17. The seedlings grew for 11 days including 5-day germination and growth at 25°C, 3-day chilling stress at 5°C and 3-day recover growth at 25°C. **Ck**. Control: maize seeds coated with formula 0; **J1**. maize seeds coated with formula 1; **J2**. maize seeds coated with formula 2; **S1**. maize seeds coated with formula 3; **S2**. maize seeds coated with formula 4; **J1S1**. maize seeds coated with formula 5; **J2S2**. maize seeds coated with formula 6. The coating agents were applied at a rate of 1 g per 5 g naked seeds for all of the treatments. For the different formulas, see the explanations in Table 1.)

No significant differences were found in the RL, SH, or RDW and SDW among the J1, J2 and control in either maize variety (Tables 3 and 4). Compared with the control, the treatment with the lower SA concentration (S1) exhibited a significantly higher RL, SH, RDW and SDW. Both J1S1 and J2S2 significantly improved the seedling growth of HC and Mo17; the most pronounced effect was observed for J2S2 (Fig. 7). In addition, the seeds coated with S2 were significantly inhibited in all of the seedling parameters compared with the control and all other treatments, irrespective of the variety (Fig. 7). By setting the control values of the seedling quality indexes to unity, the relative RL, SH, RDW and SDW values of the six treatments to the control could be calculated. J2S2 exhibited the most improved relative values in comparison to the other five coating treatments (Table 4). For Mo17, the relative SH value of J2S2 was more than two times greater than the value of the control, and the relative RDW and SDW values were also approximately twice the control values.

**Table 3:** Effect of coating agent on maize root length and shoot height under chilling stress.

Treatment	HC				Mo17			
	RL (cm)	R-RL	SH (cm)	R-SH	RL (cm)	R-RL	SH (cm)	R-SH
Control**	10.444±0.602c*	1.00	3.867±0.136d	1.00	10.984±0.257c	1.00	1.807±0.112c	1.00
J1	10.228±0.173c	0.97	3.756±0.061d	0.97	10.947±0.503c	0.99	1.751±0.114c	0.97
J2	11.211±0.404c	1.07	3.758±0.134d	0.97	11.405±0.587bc	1.04	1.560±0.079cd	0.86
S1	13.935±0.474b	1.33	5.227±0.074c	1.35	12.936±0.203b	1.18	3.109±0.228b	1.72
S2	7.080±0.049d	0.68	2.987±0.091e	0.77	6.220±0.414d	0.57	1.259±0.054d	0.69
J1S1	13.252±0.171b	1.27	6.292±0.332b	1.63	12.573±0.414b	1.14	3.249±0.270b	1.79
J2S2	15.907±0.407a	1.52	6.946±0.108a	1.80	14.648±0.450a	1.33	3.954±0.228a	2.19

RL, root length; SH, shoot height; R-RL, relative root length; R-SH, relative shoot height; HC, Huang C.

*Significant difference (α = 0.05, LSD) among treatments within the same variety.

**For additional explanations, see Table 2.

**Table 4:** Effect of coating agent on maize root dry weight and shoot dry weight under chilling stress.

Treatment	HC				Mo17			
	RDW (g/10plants)	R-RDW	SDW (g/10plants)	R-SDW	RDW (g/10plants)	R-RDW	SDW (g/10plants)	R-SDW
Control**	0.247±0.023cd*	1.00	0.128±0.002c	1.00	0.154±0.003c	1.00	0.083±0.005c	1.00
J1	0.232±0.019d	0.94	0.126±0.003c	0.98	0.130±0.003cd	0.84	0.083±0.003c	1.00
J2	0.235±0.012cd	0.95	0.126±0.004c	0.98	0.148±0.03c	0.96	0.082±0.001c	0.99
S1	0.331±0.017b	1.34	0.188±0.004b	1.47	0.180±0.007b	1.17	0.112±0.011b	1.35
S2	0.091±0.018e	0.37	0.104±0.004d	0.81	0.113±0.007d	0.73	0.027±0.004d	0.83
J1S1	0.312±0.046bc	1.26	0.192±0.005b	1.50	0.184±0.002b	1.20	0.110±0.009b	1.34
J2S2	0.462±0.027a	1.87	0.226±0.008a	1.76	0.306±0.016a	1.99	0.161±0.010a	1.94

RDW, root dry weight; SDW, shoot dry weight; R-RDW, relative root dry weight; R-SDW, relative shoot dry weight; HC, Huang C.

*Significant difference (α = 0.05, LSD) among treatments within the same variety.

**For additional explanations, see Table 2.

### Effect of coating agents containing SA-loaded P(NIPAm-*co*-BMA) on maize seedling protective enzyme activities and MDA content under chilling stress

**Fig 8:**
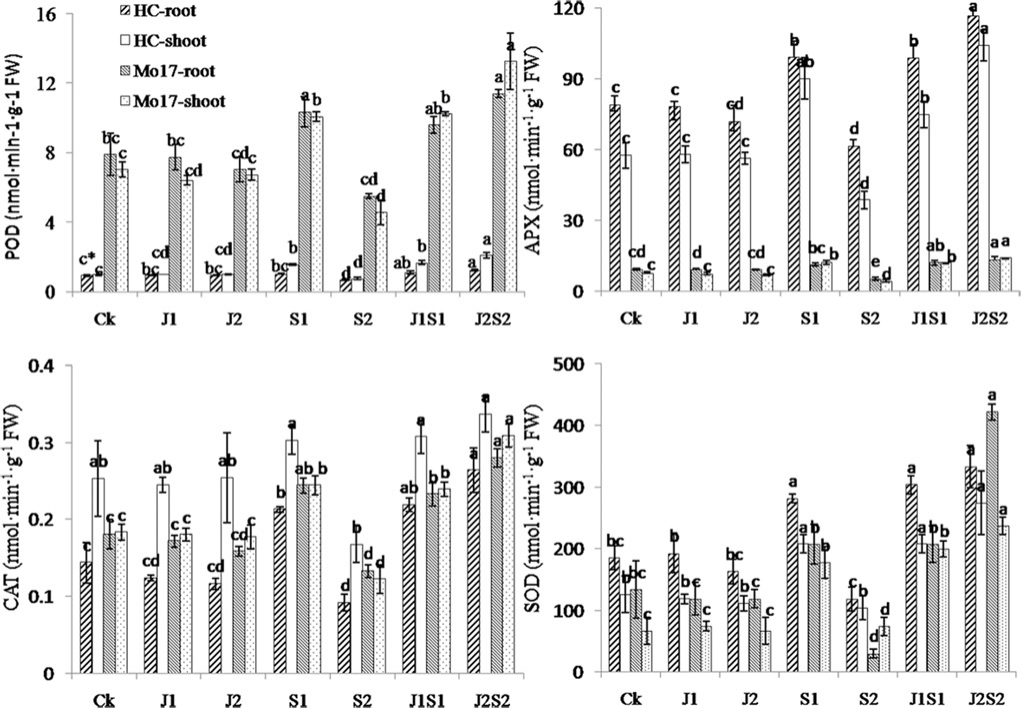
Effect of coating agent on seedling peroxidase (POD), ascorbate peroxidase (APX), catalase (CAT) and superoxidase dismutase (SOD) activities of the maize varieties Huang C (HC) and Mo17 after chilling stress. (*significant difference (a = 0.05, LSD) among treatments within the same seedling part for the same variety. Error bars represent ±S.E. For additional explanations, see Fig. 7.)

No significant differences were detected in the POD, APX, CAT or SOD activities among J1, J2 and the control in either maize variety. S1 improved protective enzyme activities in both the seedling roots and shoots, and most parameters achieved significant levels. S2 had a higher SA concentration and significantly inhibited these parameters (Fig. 8). J1S1 improved the protective enzyme activities of the HC and Mo17 seedlings, and these increased parameters reached significant levels (except the POD and SOD in the Mo17 roots and CAT in the HC shoots compared with the control). The protective enzyme activities of seeds treated by J2S2 were significantly higher than those of the control, J1and S2.

**Fig 9:**
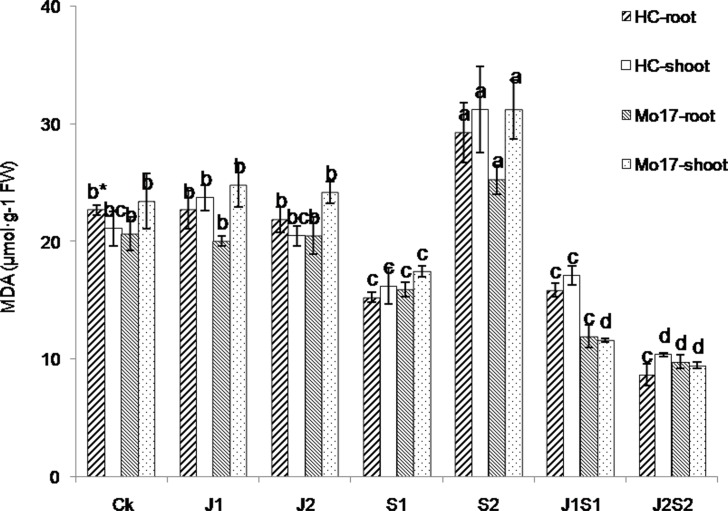
Effect of coating agent on seedling malondialdehyde (MDA) content of maize varieties Huang C (HC) and Mo17 after chilling stress. (*significant difference (a = 0.05, LSD) among treatments within the same seedling part for the same variety. Error bars represent ±S.E. For additional explanations, see Fig. 7.)

No significant differences were found in MDA content among J1, J2 and the control in either maize variety (Fig. 9). Compared with the control, the treatment with the lower SA concentration (S1) resulted in a significantly decreased MDA content, whereas S2 significantly increased the MDA content. Both J1S1 and J2S2 significantly reduced the MDA content of HC and Mo17; the most pronounced effect was observed for J2S2.

## Discussion

In the present study, a thermoresponsive P(NIPAm-*co*-BMA) hydrogel was synthesized by free-radical polymerization using a certain ratio of NIPAm monomer to BMA and BIS. Based on FTIR analysis, amides, isopropyls and esters were considered three key chemical groups in the P(NIPAm-*co*-BMA) hydrogel. Therefore, the hydrophobic-hydrophilic balance determined the swelling characteristics of the hydrogel when exposed to different external temperatures. This result is consistent with related reports on PNIPAm [23, 24]. In addition, both specific absorption peaks of P(NIPAm-*co*-BMA) and characteristic peaks of SA could be observed in the FTIR spectra of SA-loaded P(NIPAm-*co*-BMA). Therefore, the SA was successfully loaded into the hydrogel.

The LCST of the P(NIPAm-*co*-BMA) hydrogel synthesized in this study was 12°C. This value is lower than the LCST of PNIPAm (32°C). Therefore, the LCST of the hydrogel can be decreased by polymerizing NIPAm with BMA [25]. This phenomenon has also been observed in the polymerization of NIPAm with EMA (ethyl methacrylate) [26]. This decrease may occur because EMA and BMA are hydrophobic acrylate monomers, which may prevent hydrogen bonding between NIPAm amide groups and water molecules. As the temperature increased, less energy was required for the hydrogen-bonding disruption, thus contributing to the decreased LCST [26].

SA release from the P(NIPAm-*co*-BMA) hydrogel showed a stepwise response to the circulative pulse temperatures. This phenomenon was also reported by Bea *et al*. (1987) [27], who used indometacin as a model drug and observed that indometacin was released from P(NIPAm-*co*-BMA) quickly and slowly in response to temperatures of 20°C and 30°C, respectively. The different hydrogel swelling rates observed at different temperatures and the good swelling-deswelling performance may be related to the relatively loose structure of the hydrogel. The hydrogel, as a drug-delivery system, provided an ‘on-off’ model for the SA release because of its temperature-responsive pulsatile performance. In addition, the SA release quantities and modes of alteration under pulse temperatures were nearly identical in aqueous solutions with different pH values. The results suggest that the SA release was minimally influenced by pH; thus, the practical application scope of SA-loaded P(NIPAm-*co*-BMA) hydrogels can cover a wide range of soil pH values.

SA was used as the chilling resistance agent, and a SA-loaded hydrogel was used to produce an “intelligent” coating agent for coated maize seeds. An “intelligent” chilling-resistant coating treatment should not negatively influence seed germination or seedling growth. Additionally, the agent should have high efficiency in chilling resistance to protect the plant from low temperatures. With these two properties, the synthesized compound would then be an “intelligent” coating agent. When seeds or seedlings suffer from low temperature stress, negative effects on seed germination or seedling establishment can occur [28], such as excessive generation of reactive oxygen species (ROS) [29]. ROS inhibit plant growth by destroying the nucleic acid structure, retarding protein synthesis, accelerating peroxidation and damaging membrane systems [30]. However, the cooperation of protective enzymes, such as SOD, CAT, POD and APX, can eliminate ROS, maintain a homeostasis between the production and elimination of ROS and reduce the level of free radicals, resulting in less damage to the plant [31]. In addition, the changes in the cellular redox state based on the activation of various antioxidants may also induce various defense mechanisms against environmental stresses [32]. MDA is a major component of thiobarbiturate-reactive substances, and the concentration of MDA is often used as an indicator of lipid peroxidation in plant cells [33]. A higher MDA content indicates more serious damage caused by low temperature stress. The present results show that the SA-loaded P(NIPAm-*co*-BMA) hydrogel prevented damage to some extent during maize seed establishment. Appropriate doses could improve seed germination and seedling growth under low temperatures. The lack of damage may have resulted because P(NIPAm-*co*-BMA) hydrogel is nontoxic to maize seed germination and seedling growth in comparison with the control. (data compared among the J1, J2 and control treatments) and because when the seeds or seedlings suffered from chilling stress, the rapid response of the hydrogel to low temperature may have accelerated the SA release to enhance seed or seedling chilling tolerance (see Fig. 7).

SA plays an important role in the activation of resistance towards most types of abiotic stresses to alleviate injury to plants [34, 35]. In this study, the concentration of SA was crucial. If the concentration is too high, then the SA will decrease the chilling tolerance of plants [36, 37]. When the seeds were coated with 0.05 g of SA, improved seed germination, seedling growth and protective enzyme activities were observed, while the MDA concentration was reduced in seedlings. However, when 0.5 g of SA was applied, the seed germination and seedling growth were inhibited. Nevertheless, a remarkably positive effect on maize seeds and seedlings was noted when 0.5 g of SA was loaded in the P(NIPAm-*co*-BMA) hydrogel. The hydrogel could intelligently control SA release according to the external temperature fluctuations, thereby preventing excessive SA release under non-chilling stress and avoiding any resultant plant damage.

In conclusion, a P(NIPAm-*co*-BMA) hydrogel was used as a temperature-responsive carrier to realize “on-off” regulation of the release of entrapped SA in response to external temperature fluctuations. This hydrogel is a novel and intelligent method for enhancing the chilling tolerance of seed coatings. To extend this study, the efficacy of the “intelligent” thermoresponsive coating agent will be verified in the soil matrix in the growth chamber and then in the field. Additionally, the release performance of other hormones and compounds loaded in the P(NIPAm-*co*-BMA) hydrogel must be further studied to enhance maize chilling tolerance. The formula and polymerization process of the thermoresponsive hydrogel should also be improved to obtain different “on-off” regulation temperatures according to different chilling stresses of other plants.
